# General Hints on Nursing

**Published:** 1886-12-04

**Authors:** 


*(To be continued.)*

### Till the Doctor Comes.

Inquiries and Communications for this column should be addressed, "Physician," care of the Editor of THE HOSPITAL.]

### V.—GENERAL HINTS ON NURSING.

**Rules for the Sick-room.** CONSIDER in all things the comfort of the patient. If light or noise seems to annoy him, rectify the grievance as effectually as possible. Do not worry him with unnecessary questions, or press food on him when he has great distaste for it, unless you are specially told to do so by the medical man. Loud talking must on no account be permitted; but whispering is often just as cruel, as the patient may strain his attention to hear what is said. Do not discuss his symptoms before him, and especially not any bad symptoms; when you think he is asleep he may be only dozing, and it would then be gross cruelty. Avoid all unnecessary noises; do not rustle a newspaper; bring the coal up wrapped in paper, and put it on the fire with your hand. Do not allow visitors into the room indiscriminately, but only such as are likely to be quiet and helpful. Always look cheerful and pleasant before him, and, if he is low-spirited, do your best to encourage him. Empty slops outside the room, carefully clean all vessels, and keep a small quantity of some disinfectant in the bed-pan. Never leave the room without looking round to see if there is anything you have to take with you.

**Arrangement of the Sick-room.** This room, if possible, should be on the cheerful and quiet side of the house. A fireplace as a means of ventilation is most essential. The bed should not be exactly facing the window, and it should be near the centre of the bedroom, that air may get to it on all sides, and the nurse move easily round it. Screens may be placed, if necessary, so as to exclude superfluous light and draughts. All useless ornaments and articles likely to form a lodging for dust must be removed, but a daily supply of fresh flowers will tend to brighten and enliven the room. Place a small table by the bed within easy reach of the patient, and on it any drink, fruit, etc., that he may be constantly desiring. Keep the medicines handy, but all poisonous drugs should be kept carefully by themselves, and preferably under lock and key. The temperature of the room should be from 60° to 65°, except in bronchitis, croup, or other diseases, in which the doctor's orders must be taken. The thermometer should neither hang exactly over the fireplace nor in the draught from the window.

Ventilation. This is a most essential requisite, but for which probably least provision is ordinarily made. When possible, and it is very seldom not so, one of the windows should always be down an inch at the top; a screen being arranged so that there shall be no draught on the patient. Do not be afraid of night air. This fear is a popular delusion; in towns, at any rate, the night air is usually the purest. A small fire or a lamp in the grate will help greatly to purify a room. On no account burn pastilles and spray scent about a sick-room; such means only conceal the smell without purifying. If there is any smell, let it be removed by ventilation. In very cold weather, or when the patient is suffering from certain throat and lung affections, the room must be ventilated thoroughly three or four times a day, by either opening the window freely for a few minutes, and covering the patient entirely with blankets, or by first admitting fresh air into an adjoining room, and then, when it is warmed, opening the intervening door. It is seldom advisable to ventilate by means of the door; the stale air of the house, kitchen smells, and noises get in at the same time. The number of cases in which the window is better kept closed is quite infinitesimal. It is a most fearful but popular mistake that fresh air is in some way poisonous.

Washing. Unless otherwise ordered, the patient should have his body sponged over as far as practicable every morning, and the face and hands again at night. There are few diseases in which this is impracticable, if it is carefully performed, and too much of the body not exposed at one time, and it adds greatly to the patient's comfort. The teeth and mouth require especial care if he is unable to attend to them himself, or dark-coloured foul discharges gather round the teeth and gums. The nurse should in these cases dip a rag in sanitas and water, or lemon-juice and water, and carefully cleanse the teeth and gums with it, afterwards burning the rag. In some fever cases this must be done very frequently. A small piece of stick with the rag tied to it is preferred by some. Mackintoshes over the sheet to protect it whilst washing are useful, but they strike terribly cold unless first well warmed.

